# Comparison of omalizumab and mepolizumab treatment efficacy in patients with atopic and eosinophilic “Overlap” severe asthma: Biological agent preference in atopic-eosinophilic severe asthma

**DOI:** 10.1097/MD.0000000000033660

**Published:** 2023-05-05

**Authors:** Fatma Merve Tepetam, Ali Burkan Akyildiz, Şeyma Özden, Cihan Örcen, Tuğçe Yakut, Özge Atik

**Affiliations:** a University of Health Sciences, Süreyyapasa Chest Diseases and Thoracic Surgery Training and Research Hospital, Department of Immunology and Allergy, Istanbul, Turkey; b University of Health Sciences, Derince Training and Research Hospital, Kocaeli, Turkey; c University of Health Sciences, Diyarbakir Gazi Yasargil Training and Research Hospital, Diyarbakir, Turkey.

**Keywords:** atopic asthma, eosinophilic asthma, mepolizumab, omalizumab, severe asthma

## Abstract

Approximately 1-third of patients with severe asthma are candidates for both omalizumab and mepolizumab treatment. We aimed to compare the clinical, spirometric and inflammatory efficacy of these 2 biologics in atopic and eosinophilic “overlap” severe asthma patients. In our 3-center retrospective cross-sectional observational study, the data of patients who received omalizumab or mepolizumab for at least 16 weeks to treat severe asthma were examined. Atopic (perennial allergen sensitivity and total IgE level 30–1500 IU/mL) and eosinophilic (blood eosinophil counts ≥150 cells/µL in admission; or ≥300 cells/µL in the previous year) patients with asthma suitable for both biologics were included in the study. Post-treatment changes in the asthma control test (ACT) score, number of attacks, forced expiratory volume in 1 second (FEV1), and eosinophil count were compared. The rates of any biological responder patient were compared according to whether they had high eosinophil counts (≥500 cells/µL vs <500 cells/µL). Total of 181 patients data were evaluated, of the 74 atopic and eosinophilic overlap patients included in the study, 56 were receiving omalizumab and 18 were receiving mepolizumab. When omalizumab and mepolizumab treatment efficacies were compared, there was no difference in terms of the reduction in attacks and improvement in ACT. The decrease in eosinophil levels in patients in the mepolizumab arm was significantly higher than that in patients in the omalizumab arm (46.3% vs 87.8%; *P* < .001). The improvement in FEV1 was greater with mepolizumab treatment, although the difference was not significant (215 mL vs 380 mL; *P* = .053). It has been shown that having high eosinophil counts does not affect the clinical and spirometric responder patient rates for either biological condition. The success of omalizumab and mepolizumab treatment is similar in patients with atopic and eosinophilic overlap with severe asthma. However, because the baseline patient inclusion criteria are not compatible, head-to-head studies comparing both biological agents are required.

## 1. Introduction

Asthma is a common and heterogeneous disease characterized by chronic airway inflammation. It is characterized by variable airway limitations and a history of airway symptoms such as wheezing, shortness of breath, chest tightness, and cough. The duration and severity of symptoms vary.^[1]^ Symptoms can be controlled using inhalation treatment and lifestyle changes.

In contrast, severe asthma without symptom control is associated with a significant proportion of asthma-related mortality, morbidity, and economic burden.^[1–3]^ Severe asthma means that asthma is uncontrolled despite adherence to maximally optimized high-dose inhaled corticosteroid-long-acting beta-agonists (ICS-LABA) treatment and management of contributory factors, or worsens when high-dose treatment is decreased.^[1]^ Therefore, differential diagnoses should be excluded, comorbidities should be optimized, triggers should be eliminated, and patient compliance should be ensured before further treatment is planned for the diagnosis of severe asthma.^[3–5]^ The next step in planning treatment is to determine the asthma phenotype and endotype, which is particularly helpful in selecting the biological agent to be used. Biological agents show satisfactory results, especially for endotypes defined as type 2 (blood eosinophil count ≥150 cells/µL, FENO ≥20 ppb, sputum eosinophil count >2%, allergen-induced asthma, and/or steroid-dependent asthma).^[1]^ While atopic asthma, which is frequently encountered in phenotyping, shows early onset, symptom predominance, and relatively fewer eosinophilic features in many cluster analysis studies, the eosinophilic phenotype manifests with late-onset, high bronchial hyperreactivity (BHR), reversibility, and therefore, attack predominance.^[6–8]^ In a recent study 45% of patients had the eosinophilic phenotype, 50% had atopic phenotype, and 25% had phenotypic overlap.^[9]^ In the IDEAL study, 21% of patients were candidates for omalizumab, 20% for mepolizumab, and 6% (approximately 1-third) were suitable candidates for both biologics.^[10]^ However, the licensing of omalizumab, which was approved by the Food and Drug Administration in 2003, and mepolizumab, which was approved in 2015, may vary across countries. Eligibility criteria for omalizumab treatment in Turkey were age ≥ 12 years, uncontrolled symptoms, history of multiple asthma attacks despite high-dose ICS-LABA and/or leukotriene receptor antagonist treatment, forced expiratory volume in 1 second (FEV1) <80%, perennial allergen sensitivity, and total IgE level between 30 and 1500 IU/mL. The eligibility criteria for mepolizumab treatment, which has been used in our country since 2019, were as follows: age ≥18 years, peripheral blood eosinophil count ≥300 cells/µL (≥150 cells/µL in patients using long-term systemic steroids), at least 2 exacerbations in the last 1 year despite high-dose ICS-LABA and 3rd controlling agent (requiring at least 3 days of systemic corticosteroid therapy), or controlled/uncontrolled asthma under systemic steroids for at least 6 months. Patients who were candidates for mepolizumab treatment appeared to have a relatively more severe asthma.

When the mechanisms of these 2 biological agents were evaluated, omalizumab was found to be a humanized monoclonal antibody produced using recombinant DNA techniques. It is an immunoglobulin G_1_ (IgG_1_) antibody that binds free-circulating IgE (anti-IgE mAb).^[10,11]^ It binds to the IgE constant region, blocking the interaction between free IgE and high- and low-affinity IgE receptors (Fc epsilon RI receptor/FcεRI and Fc epsilon RII receptor/FcεRII, respectively) mainly on basophils and mast cells. As a result, omalizumab reduces circulating free IgE levels and downregulates FcεRI receptor expression.^[8]^ Ultimately, Omalizumab suppresses allergic inflammation by inhibiting the release of mediators from mast cells and eosinophils resulting from allergen exposure. In addition, it has been determined that Omalizumab blocks IL-2, IL-13, and GMCSF, which are involved in the activation of eosinophils, and reduces IL-5 release from peripheral mononuclear cells, thus suppressing eosinophilic inflammation.^[12]^ Omalizumab was administered subcutaneously every 2 to 4 weeks. The dose was calculated using a nomogram that used baseline serum IgE levels and body weight as the parameters. It has been shown that omalizumab provides better asthma control, reduced exacerbations, and fewer emergency room visits.^[13]^

Interleukin-5 (IL-5) is an essential cytokine for the production, migration, maturation, activation, and proliferation of eosinophils. Mepolizumab is a monoclonal humanized antibody that binds IL-5 (anti IL-5 mAb) and prevents its interaction with interleukin-5 receptor alfa (IL-5Rα). Mepolizumab decreases the recruitment and survival of eosinophils, inhibits smooth muscle proliferation and remodeling by decreasing eosinophil-derived tumor growth factor secretion, reduces tissue damage by decreasing eosinophil cationic protein and eosinophil peroxidase secretion, decreases mucus secretion and basophil activation by decreasing IL-13, and suppresses BHR by decreasing leukotriene synthesis.^[14,15]^ Looking at the clinical reflection mepolizumab reduced asthma exacerbations, improved asthma control, and reduced oral glucocorticoid requirement in this patient group.^[16–18]^

Although both biological treatments have been shown to improve asthma control and FEV1 and reduce exacerbations and eosinophils, no study has compared these 2 biological agents individually. In a review that included randomized controlled studies, patients who were candidates for overlap (i.e., candidates for both biologics) were compared, and the clinical and spirometric efficacies and side effect profiles were found to be similar. However, when patients who were candidates for omalizumab alone and mepolizumab alone were compared, the success rate of attacks was 37% higher in the group that received mepolizumab.^[19]^ In another meta-analysis, omalizumab treatment was found to be superior in terms of quality of life, but no difference was found in terms of its positive effects on asthma control and spirometry.^[20]^

Based on this information, a particular patient group met the eligibility criteria for both mepolizumab and omalizumab treatments. Ultimately, the clinician must decide on the agent to be used. In this study, we primarily aimed to retrospectively compare the clinical spirometric and inflammatory outcomes of omalizumab and mepolizumab in patients with atopic and eosinophilic severe persistent/overlap asthma. Secondary, it was aimed to compare the omalizumab and mepolizumab responder rates by considering whether the patients had high eosinophil counts (≥500 cells/µL vs <500 cells/µL).

## 2. Method

### 2.1. Study design

In our 3-center (Istanbul:115 patients, Kocaeli:44 patients, Diyarbakir:22 patients) retrospective cross-sectional observational study, the data of patients who had used omalizumab or mepolizumab for at least 16 weeks to treat severe asthma between 2012 and 2022 were examined. Over 18 years of age, we defined overlapping patients with the presence of both atopy (perennial allergen sensitivity and total IgE level 30–1500 IU/mL) and eosinophilia (blood eosinophil counts ≥150 cells/µL under systemic steroid or in admission; or ≥300 cells/µL in the previous year) who were suitable for both omalizumab and mepolizumab treatments were included in the study. (İstanbul:46 patients, Kocaeli:17 patients, Diyarbakir:11 patients) Patients with comorbid diseases such as Chronic obstructive pulmonary disease, bronchiectasis, other causes of eosinophilia (allergic bronchopulmonary aspergillosis/mucosis), eosinophilic granulomatosis with polyangiitis, malignancy, rheumatological disease, vasculitis, interstitial lung disease and patients with missing data were excluded from the study.

The study protocol was approved by the local ethics committee of the University of Health Sciences, Süreyyapaşa Chest Diseases, and the Thoracic Surgery Training and Research Hospital (approval number:160). This study was conducted in accordance with the standards of good clinical practice and the Declaration of Helsinki.

### 2.2. Patients

Patients with a confirmed diagnosis of asthma, variable respiratory symptoms, and variable airflow limitation according to the criteria specified in the global initiative for asthma guidelines were included in the study. Patients whose asthma was not controlled with high-dose ICS therapy and a second control agent, had 2 or more asthma attacks (requiring at least 3 days of systemic steroid use in the last 1 year), had a history of hospitalization for at least 1 asthma attack despite good inhaler drug compliance, correct inhaler use technique, and optimization of concomitant diseases with appropriate treatment were considered to have severe asthma. However, montelukast and/or a long-acting muscarinic antagonist were added to the treatment of patients with severe asthma before starting biologic therapy, and omalizumab or mepolizumab was started if control could not be achieved after 3 months of evaluation.

Among these patients, those over 18 years of age with a total immunoglobulin E (IgE) level of 30 to 1500 IU/mL, who had received at least 16 weeks of omalizumab (Xolair, Novartis-Switzerland) therapy, and whose perennial allergen sensitivity was determined by specific IgE (ImmunoCap; Pharmacia Diagnostics AB, Uppsala, Sweden) or skin prick test were included in the study in the omalizumab arm. Omalizumab was administered subcutaneously every 2 to 4 weeks. The dose was calculated using a nomogram that used baseline serum IgE levels and body weight as the parameters. Patients with severe asthma who had a peripheral blood eosinophil count of ≥150 cells/µL during systemic steroid therapy or on admission or ≥300 cells/µL in the previous year and who had been using mepolizumab (Nucala, Glaxosmithkline-UK) for at least 16 weeks were included in the mepolizumab arm. The mepolizumab treatment dose and dose range were 100 mg subcutaneously (sc) every 28 days (4 weeks). Among the patients in both arms, those with both allergic (perennial allergen sensitivity and total IgE level 30–1500 IU/mL) and eosinophilic (blood eosinophil counts ≥150 cells/µL during systemic steroid therapy or in admission or ≥300 cells/µL in the previous year) phenotype were included in the study by evaluating the overlap.

### 2.3. Collected data

The demographic characteristics of the patients, age at onset of asthma, duration of disease, smoking history, presence of atopy, body mass index, asthma control test (ACT) score, number of asthma attacks (requiring at least 3 days of systemic corticosteroid therapy, emergency admission, or hospitalization), FEV1 mL, FEV1%, peripheral blood eosinophil level, total IgE levels, and duration of treatment with biological agents were recorded. Although a total of 190 patients were examined, 9 patients were not included in the study due to lack of data. Seventy-four patients with atopic eosinophilic overlap was 74 (40.8%) had atopic eosinophilic overlap. Records were reviewed to evaluate the clinical, spirometric, and inflammatory parameters of omalizumab and mepolizumab. Post-treatment changes in the ACT score, number of attacks, FEV1 levels, and eosinophil counts were compared. The rates of omalizumab and mepolizumab responders were compared according to whether the patients had high eosinophil counts (eosinophil ≥500 cells/µL vs <500 cells/µL) based on changes in ACT, number of attacks, and FEV1, as indicated below.

* Responders according to ACT: An ACT increase of ≥3 points and/or an ACT score of ≥20.
* Responders according to the number of attacks: ≥50% reduction in the number of attacks.
* Responders according to FEV1: this was evaluated as an increase of ≥100 mL and/or a rate of increase of ≥10% in FEV1.

### 2.4. Statistical analysis

Statistical analyses were performed using SPSS software (version 21.0; SPSS Inc., Chicago, IL). Parametric variables are presented as means and standard deviations, while non-parametric variables are presented as medians and minimum-maximum (min-max). The numbers of cases and percentages were used as categorical variables. The chi-squared test was used to analyze categorical variables. The normality of the continuous variables was determined using Kolmogorov–Smirnov and histogram analyses. Normally distributed numerical variables were analyzed using an independent sample t-test. The Mann–Whitney U test was used to compare numerical variables that did not show a normal distribution. The difference between clinical and biological markers before and after treatment was evaluated with the Paired sample t test if it was normally distributed, and with the Wilcoxon signed-rank test if it was not. Chi-square tests were used to compare the clinical and spirometric response rates of omalizumab and mepolizumab. Statistical significance was accepted when the 2-sided *P* value was <.05.

## 3. Results

A total of 74 patients with atopic and eosinophilic overlap, 46 from Istanbul, 17 from Kocaeli, and 11 from Diyarbakir, were included in the study:56 (76%) received omalizumab, and 18 (24%) received mepolizumab. Two patients who received mepolizumab had previously switched to omalizumab. Treatment duration was longer in the omalizumab arm than in the mepolizumab arm (40 vs 13 months; *P* < .001). When the omalizumab and mepolizumab treatment arms were compared in terms of baseline characteristics, they were similar in terms of age, sex, age at asthma onset, body mass index, smoking history, and comorbidities. Patients in the omalizumab arm had a longer disease duration and higher number of attacks, whereas those in the mepolizumab arm had a higher baseline eosinophil level. There were 7 steroid-dependent patients in total, and the groups were similar in terms of both the number of patients and steroid dose used (Table 1).

**Table 1:** Baseline characteristics of the omalizumab group versus mepolizumab group.

	Omalizumab (n:56)	Mepolizumab (n:18)	*P* value
Age (yr old), mean ± SD	49.39 ± 11.48	44.39 ± 12.99	.124*
Sex, female, n (%)	48 (85.7)	14 (77.8)	.322**
Asthma onset age (yr), mean ± SD	31.73 ± 13.42	31.89 ± 11.66	.967*
Asthma duration time, (mo) median (25%–75%. percentile)	16 (10–24)	8 (6–19)	**.016** ***
Treatment time (mo), median (25%–75%. percentile)	40 (24–69)	13 (6–15)	**<.001** ***
BMI (kg/m^2^), mean ± SD	28.78 ± 6.21	29.39 ± 5.82	.715*
Smoking history, n (%) Current smoker Exsmoker Nonsmoker	3 (5.4) 22 (39.3) 31 (55.4)	0 (0) 4 (22.2) 14 (77.8)	.200**
Daily OCS^ use, n (%) OCS dose, mean (SD) mg/d	3 (5.4) 12 (6.92)	4 (22.2) 22 (7.65)	.055 .136
Comorbidities, n (%) Allergic rhinitis Nasal polyps Gastro-esophageal reflux disease NERD	50 (89.3) 10 (17.9) 15 (26.8) 8 (14.3)	15 (83.3) 7 (38.9) 6 (33.3) 1 (5.6)	.501** .065** .592** .439**
ACT, mean ± SD	11.04 ± 3.4	11.67 ± 2.99	.484*
Asthma attack, mean ± SD	7.3 ± 4.18	4.18 ± 1.95	**.031** *
FEV_1_ (mL), mean ± SD FEV1%, mean ± SD	1873 ± 702 69.4 ± 18	2091 ± 850 70.36 ± 23.8	.352* .878*
Peripheral blood eosinophils (cells/µL), median (25%–75%. percentile)	385 (300–540)	890 (600–1350)	**<.001** ***
Total IgE (IU/mL), median (25%–75%. percentile)	302 (158–526)	500 (149–835)	.144***

Bold value: Statistically significant (*P* < .05).

ACT = asthma control test, BMI = body mass index, NERD = nonsteroidal anti-inflammatory drug, OCS = oral corticosteroid.

* Independent samples t test.

** Chi square test.

*** Mann–Whitney u test.

^The mean (SD) was only for patients using daily OCS (patients not taking daily OCS were excluded).

After treatment, there was a significant decrease in attacks and eosinophil counts in both the omalizumab and mepolizumab arms, whereas a significant improvement was observed in ACT and FEV1 (Table 2). The steroid needs of steroid-dependent patients decreased by 92% in the omalizumab arm and by 85% in the mepolizumab arm; however, a statistical comparison was not performed because the number of patients was limited.

**Table 2:** Assessment of omalizumab and mepolizumab treatment efficacy.

	Omalizumab (n:56)			Mepolizumab (n:18)		
Treatment duration (mo) median (25%–75%. percentile)	40 (24–69)			13 (6–15)		*P* value
						.016*
	Pre-treatment	Post-treatment	*P* value	Pre-treatment	Post-treatment	*P* value
ACT, mean (±SD)	11.04 (3.4)	21.82 (2.21)	<.001**	11.67 (2.9)	23.3 (1.37)	<.001**
Asthma attack median (25%–75%. percentile)	6.00 (4–10)	0 (0–1)	<.001*	5.00 (4–6)	0.00 (0–1)	<.001*
FEV_1_ (mL) mean (±SD)	1870.56 (708.09)	2189.44 (714.70)	<.001**	2091.36 (850.66)	2581.43 (770.393)	<.001**
Peripheral blood eosinophils (cells/µL), median (25%–75%. percentile)	385 (300–540)	200 (100–395)	<.001*	890.00 (600–1350)	88 (50–260)	<.001*

ACT = asthma control test, FEV_1_ = forced expiratory volume in 1 second.

* Wilcoxon t test.

** Paired samples t test.

**Figure 1:**
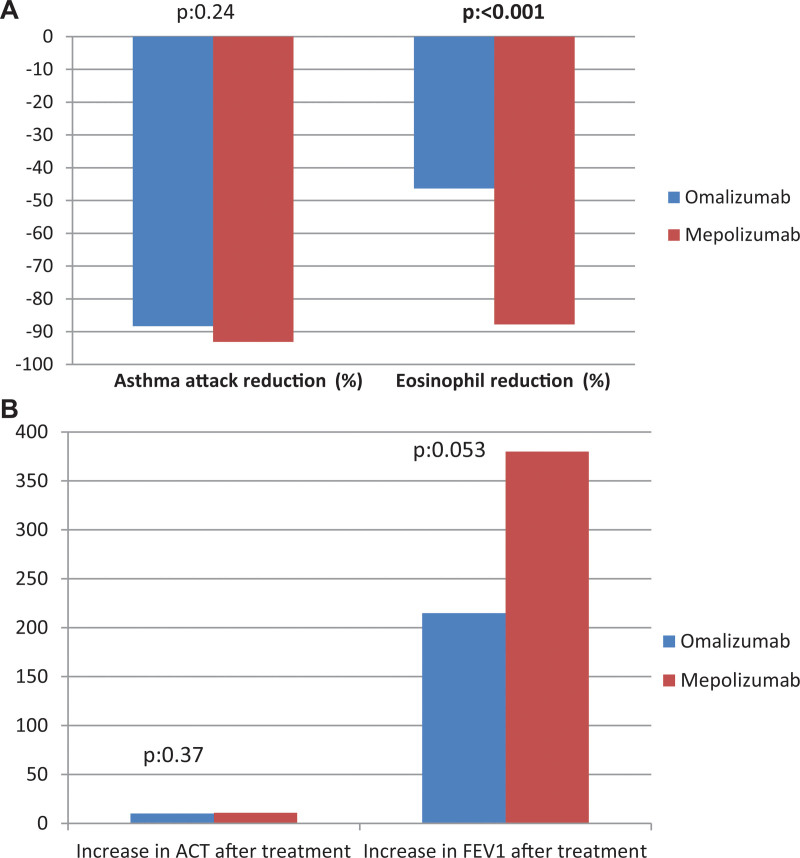
(A) Comparison of the reduction of asthma attacks and eosinophil levels after omalizumab and mepolizumab treatment. (B) Comparison of improvements in ACT and FEV1 after omalizumab and mepolizumab treatment. ACT = asthma control test, FEV1 = forced expiratory volume in 1 second.

When omalizumab and mepolizumab treatment efficacies were compared, there was no difference in terms of reduction in attacks (88% vs 93%, respectively; *P* = .24). The decrease in eosinophil levels of the patients in the mepolizumab arm was significantly higher than that in the omalizumab arm (46.3% vs 87.8; *P* < .001) (Fig. 1A). Although the improvement in ACT (10.8 vs 11.7; *P* = .37) was similar in both groups, the improvement in FEV1 was higher in favor of mepolizumab, although the difference was not statistically significant (215 mL vs 380 mL; *P* = .053) (Fig. 1B).

**Figure 2:**
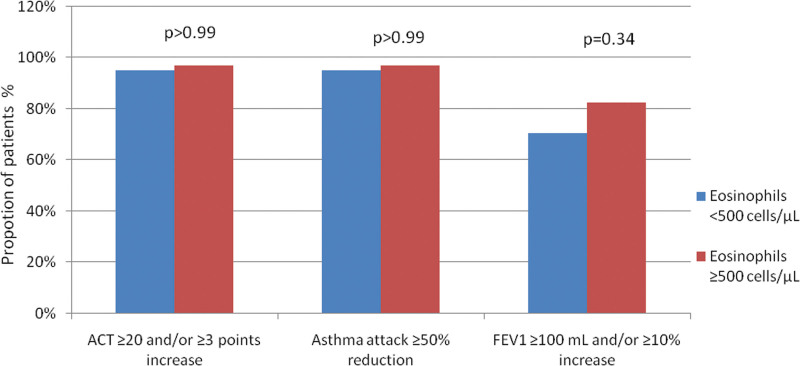
Comparison of percentage of responder patients according to eosinophilic status (<500 vs ≥500); based on increase in ACT (≥20 and/or ≥3 points), reduction of asthma attacks (≥50%), increase in FEV1 (≥100 mL and/or ≥10%). ACT = asthma control test, FEV1 = forced expiratory volume in 1 second.

All patients in the omalizumab and mepolizumab arms were found to have similar clinical (ACT responders: 95% vs 97%; *P* > .99, attack responders: 95% vs 97%; *P* > .99) response rates when taking an eosinophil cutoff value of 500 (eosinophil <500 cells/µL or eosinophil ≥500 cells/µL). However, when the responder rate was compared based on FEV1, although it was not significant, it was found to be higher in favor of the eosinophilic group (70.4% vs 82.6%; *P*: .34) (Fig. 2).

## 4. Discussion

In our retrospective cross-sectional observational study comparing the efficacy of omalizumab and mepolizumab treatment in atopic eosinophilic overlap patients, we found that while patients in the mepolizumab arm had higher eosinophil levels at baseline, those in the omalizumab treatment arm had a longer disease duration and more attacks. Significant clinical and spirometric improvements were observed in patients in both arms after treatment, whereas a significant decrease was observed in eosinophil levels. However, when both biologic agents were compared in terms of treatment efficacy, the clinical efficacy was similar, whereas the decrease in eosinophil levels was significantly higher in the mepolizumab arm, and the improvement in FEV1 was greater in favor of mepolizumab, although the difference was not statistically significant. After omalizumab or mepolizumab treatment, the clinical and spirometric responder patient rates were similar between the high and low eosinophil levels groups.

Severe asthma is a heterogeneous disease, with several distinct phenotypes. According to the global initiative for asthma guidelines, the rate of atopic-eosinophilic overlap in patients who are candidates for both omalizumab and mepolizumab therapies targeting the type 2 endotype was reported to be approximately 1-third, which was slightly higher (40.8%) in our study. However, when patients eligible for omalizumab were evaluated in the IDEAL study, an observational cohort study involving 6 countries showed a high degree of variability in the number of overlapping patients who were also eligible for mepolizumab treatment (35% vs 73%, respectively; Australia/Canada/United States of America and the European Union).^[1,10]^ The most important reason for this is the application of different criteria from country to country, including parameters such as the number of attacks, asthma control, and FEV1 in biological agent licensing. In our country, the presence of more than 1 severe attack is required for omalizumab, which has been under reimbursement since 2008, and for mepolizumab treatment, which has been under reimbursement since 2019. Additionally, patients with frequent symptoms and low lung function (FEV1 <80%) are required to initiate omalizumab treatment. Therefore, it may have been determined that patients in the omalizumab arm tended to have lower baseline FEV1 levels. In addition, since there was no biological agent treatment in our country until 2008, and there were no other options other than omalizumab in the following 10 years, it can be explained that the patients in the omalizumab arm had a longer disease duration and more attacks at baseline. In the WATCH study, in which patients in the Wessex AsThma CoHort study were retrospectively examined and the phenotypic characteristics of patients who were candidates for omalizumab and mepolizumab treatment were analyzed, omalizumab response was found to be independently associated with more attacks at baseline. However, it is less associated with Acute Healthcare Encounters (emergency department or hospital admissions).^[21]^ In our study, the number of attacks was higher in the omalizumab arm at baseline than that in the mepolizumab arm (7.3 vs 4.18; *P* = .031). However, in our study, we defined all emergency admissions, hospitalizations, and severe attacks requiring systemic steroids as “attacks. ” In the same study, patients who were candidates for mepolizumab were late-onset elderly patients with more eosinophilia and nasal polyps at baseline, and the treatment response was associated with better asthma control at baseline. Our patients in the mepolizumab arm also had higher eosinophil levels at baseline; however, their age at asthma onset, presence of nasal polyposis, and ACT scores were similar to those in the omalizumab arm, which may be due to the selection of an atopic eosinophilic overlap patient population.

No head-to-head comparative studies have been conducted on the efficacies of omalizumab and mepolizumab. In a systematic review that included the MENSA study in the Mepolizumab arm, the INNOVATE study, and the study by Chanes et al in the omalizumab arm, the efficacy was found to be similar in the overlap group, while in this systematic review (where the EXTRA study was also included) in which treatment efficacy was indirectly compared in patients who were candidates for omalizumab or mepolizumab (trial group), the success of reducing attacks was evaluated in favor of mepolizumab.^[19]^ In another post hoc analysis of the MENSA and MUSCA studies that compared the efficacy of mepolizumab with placebo in patients with asthma who were eligible and ineligible for omalizumab treatment, the clinical and spirometric efficacies were similar.^[22]^ In a very recent posthoc analysis of the REALITY-A study, which is the real-life data of mepolizumab, the efficacy was found to be similar in the overlap endotype patients who were also suitable for omalizumab.^[23]^ Similar to these studies, in our study, the effect of reducing attacks in patients was similar in both treatment arms (omalizumab:88% vs mepolizumab:93%, *P* = .24) and was consistent with other large-scale real-life studies (EXPERIENCE study for omalizumab:89.9%; French ATU for mepolizumab: 86%).^[24,25]^ In a real-life study of mepolizumab, eosinophil counts were reduced by 87% in the ATU study and 83% in the REALITY study, similar to the results of our study.^[25,26]^ In a recent study of omalizumab treatment, eosinophils were reduced by 42%, similar to the results of our study.^[27]^ In addition, 2 patients with persistent eosinophilia despite omalizumab treatment were evaluated in the mepolizumab arm because we switched to mepolizumab treatment. While the clinical efficacy was similar in the post hoc analysis of the MENSA and SIRIUS studies, which compared the patients who used Omalizumab before mepolizumab treatment and those who did not, in the MENSA study, it was found that the improvement in FEV1 was higher in patients who did not use omalizumab before. This was thought to be related to the inexplicable improvement in FEV1 after placebo treatment in patients who previously received omalizumab.^[28]^ In our study, in 2 patients who switched from omalizumab to mepolizumab, FEV1 improved and attacks decreased, as in patients who were directly treated with mepolizumab. Although the level of improvement in FEV1 was not statistically significant in our study, the improvement in the mepolizumab arm was higher (215 mL vs 380 mL; *P* = .053) can be explained by the fact that patients in the mepolizumab arm were more eosinophilic. In the ADEPT cluster analysis, the eosinophilic group was found to have high reversibility and BHR.^[8]^ The PROSPERO trial showed a better improvement in FEV1 (80 mL vs–10 mL) in the eosinophilic group after omalizumab treatment.^[29]^ This shows that these patients with high eosinophil levels are more reversible with treatment. While the improvement in FEV1% was 8.7% in the EXPERIENCE study, which is the comprehensive real-life data with omalizumab, it was 17% (340 mL) in the real-life data mepolizumab study conducted in Italy.^[24,30]^ A recent study of omalizumab reported that the eosinophilic phenotype had a higher response rate to treatment.^[31]^ Although our study showed that being eosinophilic did not affect the clinical and spirometric responder patient rates in either treatment arm, the spirometric response was evaluated in favor of the eosinophilic patient population.

## 5. Conclusion

We conducted 3-center real-life study comparing the efficacy of omalizumab and mepolizumab in both atopic and eosinophilic “overlap” severe asthma patients. Although the success of omalizumab and mepolizumab treatments in improving asthma control and reducing exacerbations is similar in atopic eosinophilic severe asthma patients, in whom we encounter more than 1-third of patients in practice, the rate of suppression of eosinophilic inflammation is in favor of mepolizumab. Although the improvement in FEV1 was not statistically significant, it may favor mepolizumab treatment, particularly in patients with eosinophil dominance. However, because the baseline patient inclusion criteria are not compatible, head-to-head comparative studies with larger numbers of patients are needed.

## Author contributions

**Conceptualization:** Fatma Merve Tepetam, Şeyma Özden.

**Data curation:** Fatma Merve Tepetam, Ali Burkan Akyildiz, Şeyma Özden, Cihan Örcen, Tuğçe Yakut.

**Formal analysis:** Fatma Merve Tepetam, Cihan Örcen.

**Investigation:** Fatma Merve Tepetam, Ali Burkan Akyildiz.

**Methodology:** Fatma Merve Tepetam, Şeyma Özden.

**Project administration:** Fatma Merve Tepetam.

**Resources:** Fatma Merve Tepetam, Cihan Örcen, Tuğçe Yakut.

**Software:** Fatma Merve Tepetam, Tuğçe Yakut.

**Supervision:** Fatma Merve Tepetam, Özge Atik.

**Validation:** Fatma Merve Tepetam, Ali Burkan Akyildiz, Şeyma Özden, Cihan Örcen, Tuğçe Yakut, Özge Atik.

**Visualization:** Fatma Merve Tepetam, Şeyma Özden.

**Writing – original draft:** Fatma Merve Tepetam, Ali Burkan Akyildiz.

**Writing – review & editing:** Fatma Merve Tepetam, Özge Atik.
